# Rapid Multiplex Strip Test for the Detection of Circulating Tumor DNA Mutations for Liquid Biopsy Applications

**DOI:** 10.3390/bios12020097

**Published:** 2022-02-04

**Authors:** Panagiota M. Kalligosfyri, Sofia Nikou, Sofia Karteri, Haralabos P. Kalofonos, Vasiliki Bravou, Despina P. Kalogianni

**Affiliations:** 1 Department of Chemistry, University of Patras, Rio, 26504 Patras, Greece; pkalligosfyri@gmail.com; 2 Department of Anatomy-Histology-Embryology, Medical School, University of Patras, Rio, 26504 Patras, Greece; sonikou@upatras.gr; 3 Division of Oncology, Department of Internal Medicine, University Hospital of Patras, Rio, 26504 Patras, Greece; skarteri@gmail.com (S.K.); kalofonos@upatras.gr (H.P.K.)

**Keywords:** colorectal cancer, KRAS, lateral flow assay, dipstick, biosensor, gold nanoparticles

## Abstract

In the era of personalized medicine, molecular profiling of patient tumors has become the standard practice, especially for patients with advanced disease. Activating point mutations of the KRAS proto-oncogene are clinically relevant for many types of cancer, including colorectal cancer (CRC). While several approaches have been developed for tumor genotyping, liquid biopsy has been gaining much attention in the clinical setting. Analysis of circulating tumor DNA for genetic alterations has been challenging, and many methodologies with both advantages and disadvantages have been developed. We here developed a gold nanoparticle-based rapid strip test that has been applied for the first time for the multiplex detection of KRAS mutations in circulating tumor DNA (ctDNA) of CRC patients. The method involved ctDNA isolation, PCR-amplification of the KRAS gene, multiplex primer extension (PEXT) reaction, and detection with a multiplex strip test. We have optimized the efficiency and specificity of the multiplex strip test in synthetic DNA targets, in colorectal cancer cell lines, in tissue samples, and in blood-derived ctDNA from patients with advanced colorectal cancer. The proposed strip test achieved rapid and easy multiplex detection (normal allele and three major single-point mutations) of the clinically relevant KRAS mutations in ctDNA in blood samples of CRC patients with high specificity and repeatability. This multiplex strip test represents a minimally invasive, rapid, low-cost, and promising diagnostic tool for the detection of clinically relevant mutations in cancer patients.

## 1. Introduction

Personalized medicine is based on recommendations according to genomic “drivers” of tumorigenesis [1]. Cancer develops through a multistage process, with the accumulation of somatic mutations and genetic alterations leading to increased cell proliferation and tumorigenesis [2]. Such mutations are known as “drivers’’ and are crucial for monitoring disease progression and resistance to targeted therapeutic agents [3,4]. Amongst known driver mutations, activating mutations in the KRAS or KRAS2 (Kirsten rat sarcoma virus 2 homolog) oncogene are found in several malignancies including colorectal cancer (CRC) [5,6,7,8]. Activating KRAS mutations are point mutations mainly affecting KRAS amino acid residues 12, 13, and 61, which reduce the intrinsic KRAS and GTPase activating protein–promoted GTP hydrolysis [9,10]. KRAS mutations occur early in the development of colorectal cancer (CRC) and are strongly associated with resistance to therapies [10,11]. CRC is the second most common cause of cancer-related mortality in Europe [12]. About 40% of CRC cases are KRAS-mutant-related, meaning that they cannot benefit from EGFR-targeted therapies [13].

In clinical practice, the majority of molecular profiling tests, including KRAS mutation detection, are performed in tissue biopsies. However, excision regions do not sufficiently depict intratumoral heterogeneity, and in some cases, tissue biopsy is not feasible. Liquid biopsy, which mainly includes analysis of cell-free DNA (cfDNA), represents a convenient real-time tool for mutational profiling in a minimally invasive manner. cfDNA is double-stranded, fragmented extracellular DNA released from normal and cancer cells through cell death (apoptosis or necrosis) or by active secretion with extracellular vesicles (exosomes and prostasomes) into the bloodstream. Plasma circulating tumor DNA (ctDNA) that originates specifically from tumors represents a small fragment (<1.0%) of the total cell-free DNA (cfDNA) and is only identified via the detection of cancer-related mutations [14,15]. Several factors, such as the tumor type, stage, and burden, as well as the tumor proliferation rate and turnover, affect the amount of ctDNA in body fluids [16,17,18]. Moreover, the average size of ctDNA ranges from small fragments of 70–200 bp which are secreted due to cellular apoptosis to longer fragments of 200 bp–21 Kbp generated by necrosis. Thus, the extremely low abundance of ctDNA, especially in early cancer stages, along with its high fragmentation, render ctDNA a challenging analyte [19,20]. The analytical methods reported for ctDNA detection include PCR-based techniques (such as digital PCR, methylation-specific PCR, and real-time PCR), next-generation sequencing (NGS), mass spectrometry, and DNA microarrays. However, many of these techniques have drawbacks in ctDNA analysis or do not meet the requirements of a diagnostic tool applicable in the clinical setting. This is due to attributes including low detectability and specificity, high cost of analysis and expensive instrumentation, long analysis time, and extensive sample pretreatment [21,22,23,24]. Given the feasibility of using ctDNA in tracking and monitoring tumor dynamics and resistance to therapy, the use of ctDNA as a marker for detecting driver mutations needs to be further developed. KRAS driver mutations in codon 12 or 13 mutations are a major predictive biomarker of poor response to therapy in patients with CRC [10,25]. Therefore, rapid and precise identification of KRAS mutations in ctDNA is required for improving the response rate and survival in CRC patients [25]. Strip-type rapid tests, and biosensors in general, represent excellent candidates for liquid biopsy applications [26]. Various biosensors have been developed so far for ctDNA detection. However, only a few reports present applications for ctDNA analysis in patients’ blood samples.

Herein, we have developed a rapid multiplex strip test that comprises of a gold-nanoparticle-based optical DNA biosensor for KRAS screening in cancer. Based on a previous developed flow strip assay for detecting KRAS mutations [27], the current multiplex strip has been applied, for the first time, for multi-analyte liquid biopsy applications. The wild-type KRAS and three major single-point KRAS mutations (G12D, G12A, G12V) were simultaneously detected in cfDNA/ctDNA with a single strip test. The strip-type DNA biosensor was tested and optimized in synthetic DNA targets, in colorectal cancer cell lines, in tissue samples and was finally applied to blood-derived cfDNA from healthy individuals and ctDNA from patients with advanced colorectal cancer.

## 2. Materials and Methods

### 2.1. Reagents and Apparatus

Polymerase chain reactions (PCR) were performed using the HotStarTaq Master Mix Kit (Qiagen, Hilden, Germany). Sections from FFPE tissue samples were cut with Accu-cut SRM 200 Rotary Microtome (The Netherlands, Europe, BV). The Nucleospin DNA FFPE XS kit used for tissue DNA extraction was obtained from Macherey-Nagel (Düren, Germany). All synthetic oligonucleotides were purchased from Eurofins Genomics (Εbersberg, Germany) (Table 1). All other reagents and apparatus used were previously reported [27].

### 2.2. Cell Lines and Clinical Samples

Human colorectal cancer cell lines, namely Caco2 and LS174T, were used in the present study. The human colorectal cancer cell lines were obtained from the American Type Culture Collection (ATCC, Manassas, Virginia USA). The mutant KRAS cell line LS174T (c.35G>A, p.G12D) and the wild-type KRAS cell line Caco2 were cultured and harvested as previously described [27].

The study included formalin-fixed paraffin-embedded (FFPE) tissue samples from three CRC patients that were retrieved from the archives of the Department of Pathology, University Hospital of Patras, Greece and peripheral blood samples from four healthy individuals and five CRC patients prior to or close to the initiation of chemotherapy treatment. All patients were treated at the Division of Oncology, Department of Medicine, Medical School, University of Patras and had known KRAS mutations detected in FFPE tissue samples by next-generation sequencing (NGS) (Ion Gene Studio S5 Prime System, Thermo Fisher Scientific) using the Ion AmpliSeq NGS Panel (Thermo Fisher Scientific). Relevant information was retrieved from the patients’ oncology records. Patient characteristics are summarized in Table S1.

Blood samples were obtained after written informed consent, and the study has been approved by the Ethics and Scientific Committee of the University General Hospital of Patras, Greece (Protocol Number 680/15.10.2019) and by the Institutional Ethics & Research Committee of the University of Patras, Greece (Protocol Number 86436/14.10.2019), in strict compliance with the ethical standards of the institutional and/or national research committee and the 1964 Declaration of Helsinki and its later amendments.

### 2.3. DNA Extraction from Cell Lines and Tissue Samples

DNA was extracted from cell lines using the NucleoSpin DNA Rapid Lysis kit, according to manufacturer’s instructions, after washing of the cell pellet with 1 × PBS, pH 7.4. DNA was also isolated from formalin-fixed paraffin-embedded (FFPE) tissue samples using the NucleoSpin DNA FFPE XS kit. All extractions were performed using 10 μm sections (3 sections from each FFPE block). All sections were initially deparaffinized using xylene (60 °C) and hydrated in descending alcohol series. DNA was then extracted from the FFPE tissue kit, following the manufacturer’s instructions. Finally, the purity and concentration of isolated DNA was determined by UV spectrometry at 260/280 nm (Table S2).

### 2.4. Cell-Free DNA (cfDNA) Extraction from Blood Samples

CfDNA was extracted from the plasma of blood samples. The plasma was carefully separated from the pellets by centrifugation for 10 min at 2500 rpm, at room temperature. The isolation of cfDNA from plasma samples was performed using the NucleoSpin Plasma XS kit, according to the manufacturer’s instructions. The cfDNA was obtained by an elution step with 20–30 μL of the provided elution buffer and centrifugation at 11,000 g for 30 s. The purity and the concentration of the isolated cfDNA was measured with the NanoDrop 1000 spectrophotometer (Table S2).

### 2.5. Preparation of Streptavidin-Conjugated Gold Nanoparticles (SA-AuNPs)

Prior to the conjugation reaction, the pH of the AuNPs solution was adjusted to 6.0 with 10 mM phosphate buffer, pH 6.8. An amount of 1.5 μg of streptavidin was then mixed with 200 μL of gold nanoparticles and the mixture was incubated for 2 h at room temperature, in the dark, with frequent stirring. After the incubation step, BSA was used as blocking agent at a final concentration of 10 g/L. The mixture was incubated for 30 min at room temperature. A 20 min centrifugation step at 3300× g was performed for the collection of the SA-functionalized AuNPs. The SA-AuNPs conjugates were finally reconstituted in 20 μL of proper storage buffer (1 × PBS pH 7.4, 1% BSA, 0.25% sucrose, 1% Tween-20, 0.05% NaN_3_) and stored at 4 °C for further use.

### 2.6. Synthesis of Functionalized Microspheres

Four different sets of functionalized carboxylated polystyrene microspheres were prepared. The four sets corresponded to the normal allele and the three single-point mutations of the KRAS gene that were examined in the present work. Each set was coupled to a specific oligonucleotide sequence (anti-tag sequence) through an amino group at its 5′ end as previously reported [28] but with some modifications. Briefly, a mixture of 12.8 μL of the carboxylated polystyrene microspheres and 125 μL of MES buffer, pH 4.5, was centrifuged for 2 min at 15,700× *g*, and the supernatant was discarded. The microspheres were resuspended in 40 μL MES buffer, pH 4.5, and sonicated for 2 min. An amount of 400 pmol of each anti-tag sequence was separately conjugated to the microspheres, using 1.25 μL of a fresh EDC, 0.4 mg/μL. The coupling reaction was held in the dark and at room temperature for 30 min with occasionally stirring. Then, the addition of the same amount of EDC was repeated. After incubation, the suspension was centrifuged for 2 min at 15,700× *g*, after the addition of 2 μL of 10% Tween-20, to collect the conjugated microspheres. The prepared anti/tag-microspheres were then washed two times with 100 μL of 0.2% Tween-20 in 1 × TE buffer, redispersed in 100 μL of 1 × TE buffer (pH 8.0), and kept at 4 °C until use.

### 2.7. KRAS Gene Amplification

The KRAS gene at exon 12, that contained the single-point mutations of interest, was amplified by PCR at a final volume of 25 μL. The PCR reaction pool contained 1 × HotStarTaq Master Mix (with 1.5 mM MgCl_2_), KRAS primers at a concentration of 0.6 μM and 100 ng of isolated DNA. The conditions of the reaction were: a first step of denaturation at 95 °C for 15 min that was followed by 35 cycles of 95 °C for 30 s, 55 °C for 30 s and 72 °C for 30 s, and a final extension step at 72 °C for 10 min. The PCR products were then electrophorized in a 2% agarose gel, using ethidium bromide for DNA staining. The PCR products were finally quantified by densitometric analysis using the open-source ImageJ image processing and gel analyzer tool by National Institutes of Health (NIH) (Bethesda, Maryland, USA) and Laboratory for Optical and Computational Instrumentation, University of Wisconsin (Wisconsin, USA).

### 2.8. Multiplex Primer Extension Reaction (PEXT) for Single-Point Mutation Discrimination in ctDNA

A multiplex primer extension (PEXT) reaction using four specific primers for the tested polymorphisms of the KRAS gene was conducted. The reaction (20 μL) consisted of 1 × Vent (exo-) buffer with 1.5 mM MgCl2, 2.5 μM each of the dNTPs except from dCTP, 2.5 μM of biotin-dCTP, 0.05 μM of each of the specific primers, 0.5 U of the DNA polymerase Vent (exo-), and 200–400 fmol of each PCR product. The PEXT reaction was performed as follows: denaturation at 95 °C for 3 min, followed by 25 cycles at 95 °C for 15 s, at 58 °C for 10 s, and at 72 °C for 15 s.

### 2.9. Fabrication of the Multiplex Rapid Strip Test

**Figure 1:**
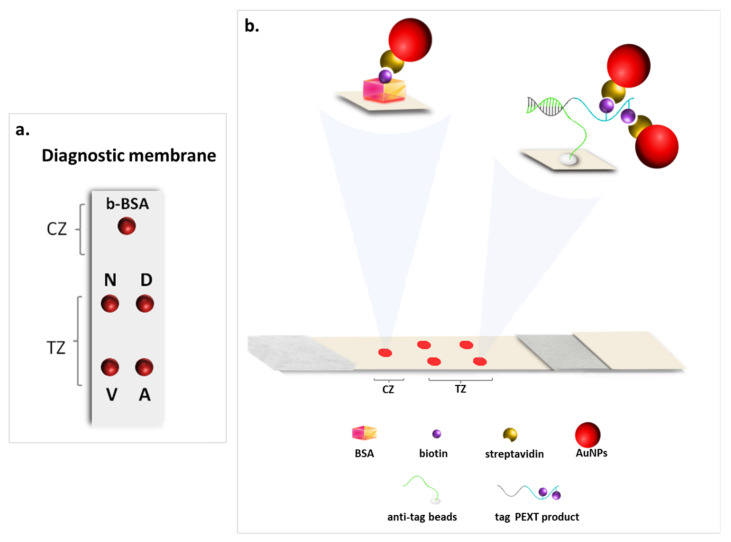
(**a**) Layout of the test and the control spots on the diagnostic membrane of the multiplex strip test. (**b**) The principle of the multiplex strip test. b-BSA: biotinylated BSA, N: normal allele, D: G12D mutant allele, V: G12V mutant allele, A: G12A mutant allele, TZ: test zone, CZ: control zone, AuNPs: gold nanoparticles.

The strip test consisted of four parts: an immersion pad, a conjugate pad, a nitrocellulose membrane, and an absorbent pad with a total size of 5 mm × 70 mm. The four parts were welded onto a plastic adhesive backing pad with overlapping ends for continuous flow. Biotinylated BSA (b-BSA) (Supplementary Material) was deposited onto the membrane (0.5 μL of 50 ng/μL), in order to construct the control zone of the strip, as an assurance of the functionality of the test. Secondly, a volume of 1 μL of each of the four sets of the microspheres coupled to the four anti-tag sequences were also placed onto the membrane, in order to form four spatially distinct test spots as presented in Figure 1. The spots were then dried for 10 min at room temperature, and the strip was ready for use.

### 2.10. Multi-Allele Detection by the Rapid Strip Test

The PEXT products were denatured by heating at 95 °C for 3 min and placed immediately on ice for 2 min. A 5 μL aliquot of the denatured PEXT products was applied onto the conjugate pad of the strip just above an 8 μL aliquot of prepared streptavidin-gold nanoparticles (SA-AuNPs) conjugates (Supplementary Material) that were pre-deposited in the same pad. The strip was then immersed into 300 μL of the developing solution that consisted of 4 × SSC pH 7.0, 1.5% glycerol, 1% Tween, 1% BSA and 0.5% SDS. The strip was removed from the solution after 15 min for visual detection of the PEXT products. Finally, images of the strips were acquired by a conventional scanner.

## 3. Results

We have developed a rapid multiplex strip test based on gold nanoparticles for the detection of gene mutations in circulating tumor DNA for non-invasive liquid biopsy applications. The homo sapiens KRAS oncogene (GTPase (KRAS), transcript variant X2, mRNA—Accession number: XM_011520653), mutations of which guide treatment decisions in colorectal cancer (CRC), was used as a model gene. The proposed strip test was applied, for the first time, for the simultaneous detection of the normal allele and three major single-point mutations of the KRAS gene in cell-free DNA and/or circulating tumor DNA from peripheral blood samples. These mutations are located in exon 12, namely G12D (35G>A), G12V (35G>T), and G12A (35G>C). Optimization parameters involved samples that consisted of (*i*) four single-stranded synthetic DNA targets corresponded to the four KRAS alleles (Table 1) and also DNA isolated from (*ii*) cell lines expressing the wild-type and the mutated (G12D) KRAS gene and (*iii*) FFPE tissue samples from CRC patients carrying known KRAS mutations. After optimization, the method was applied for the genotyping of the KRAS gene in cell-free DNA and circulating tumor DNA in blood samples from healthy individuals and CRC patients with known mutations.

The protocol included: (a) DNA or cfDNA isolation, (b) PCR of the exon 12 of the KRAS gene, (c) multiplex allele-discrimination reaction by a multiplex primer extension (PEXT) reaction, and (d) a multiplex strip test for alleles detection. The size (171 bp) and the amount of the PCR products were estimated by a 2% agarose gel electrophoresis with subsequent ethidium bromide staining (Figure S1). After amplification, each PCR product was subjected to a multiplex PEXT reaction in the presence of four allele-specific PEXT primers that were complementary to the wild-type allele and the three mutations of the KRAS gene exon 12. The primers used in PEXT reaction had the same sequence and differed only in two points: (*i*) they carried a different base at the 3′ end, complementary to each mutation and the normal allele, respectively; and (*ii*) they carried different 24-bp sequences (tag sequences) at their 5′ ends, which were complementary to the four anti-tag sequences attached to the four sets of microspheres, in order to capture the PEXT products onto the strip. The PEXT primers were then elongated only if they were fully complementary to the sequence of the KRAS allele by a special DNA polymerase that lacks the 3′ → 5′ proofreading exonuclease activity. Biotin moieties were inserted into the PEXT products during the polymerization using biotinylated dCTP. The PEXT products were finally applied onto the strip after being heat denatured for a few minutes.

The diagnostic membrane of the multiplex strip test consisted of a control (CZ) and a test zone (TZ). The TZ reveals the presence of the target in the sample, while the CZ ensures the proper function of the strip test. For the formation of the CZ, biotin was immobilized on the nitrocellulose membrane by deposition of b-BSA. A first red spot was formed at the CZ on the upper site of the membrane, as the excess of SA-AuNPs was bound there through biotin–streptavidin interactions. The TZ of the strip consisted of four test spots of the four different sets of the anti-tag-modified microspheres that were pre-deposited and were spatially discrete on the membrane, in order to capture the PEXT products. The arrangement of the test spots on the membrane was as follows (from the top to the bottom of the membrane): normal and the G12V (35G>T) alleles on the left and the G12D (35G>A) and the G12A (35G>C) alleles on the right side (Figure 1a). Moreover, an 8 μL aliquot of the SA-AuNPs conjugates and a volume of 5 μL of the heat-denatured multiplex PEXT products were deposited on the conjugate pad of the strip, and the strip was then immersed into a microcentrifuge tube containing the developing solution. All the reagents deposited on the conjugated pad were entrained upwards by the developing solution though capillary action. The products from the PEXT reaction were captured at the TZ due to tag/anti-tag hybridization, between the tag tail of the PEXT primers and the anti-tag sequences that were coupled to the immobilized microspheres. The hybridization was finally visually detected by the accumulation of the SA-AuNPs at the same spots through the interaction of streptavidin with the biotin moieties of the PEXT products, forming up to four visual red spots (Figure 1b). The excess of SA-AuNPs was directed to the CZ of the strip, forming another red spot. The visual detection by naked eye was completed after 10–15 min.

The successful preparation of SA-AuNPs conjugates was tested by analyzing the conjugates with two strip tests with immobilized biotinylated and non-biotinylated BSA, respectively. As observed from Figure S2 in the Supplementary Material, the conjugates were specifically captured; thus, a red spot was formed only on the strip on which b-BSA was immobilized due to streptavidin-biotin interaction.

The whole method was first developed and optimized using four 60-bp single-stranded synthetic DNA targets that corresponded to the four analyzed alleles (Table 1). Optimization studies involved the cycling conditions of the PEXT reaction, as well as the performance of the strip test, in order to increase the signal density and eliminate the non-specific interactions at the test zone of the strip. In more detail, the specific parameters studied were: the cfDNA isolation procedure for higher yield and purity, the conditions of the primer extension reactions, and the process of SA-AuNPs preparation that included the concentration of the reagents and the pH of the coupling reaction, as well as the final reconstitution buffer of SA-AuNPs. The composition of the developing solution was also tested, regarding the use of surfactants to minimize the non-specific bindings and the concentration of glycerol to provide the required time for efficient hybridization. Finally, the sample volume applied onto the strip test was optimized. For this purpose, different sample volumes (1–10 μL) that contained only the normal KRAS allele were applied onto the multiplex strip test. As observed from Figure S3 in the Supplementary Material, the optimum sample volume was 5 μL, which gave the strongest signal with high specificity.

The analytical performance of the strip test was also evaluated by assessing the detectability of a biotinylated singe-stranded DNA (ssDNA) (b-dA_30_). The results are presented in Figure S4. As low as 50 amol of ssDNA were detectable with the naked eye with the rapid strip test.

### 3.1. Synthetic DNA Targets

**Figure 2:**
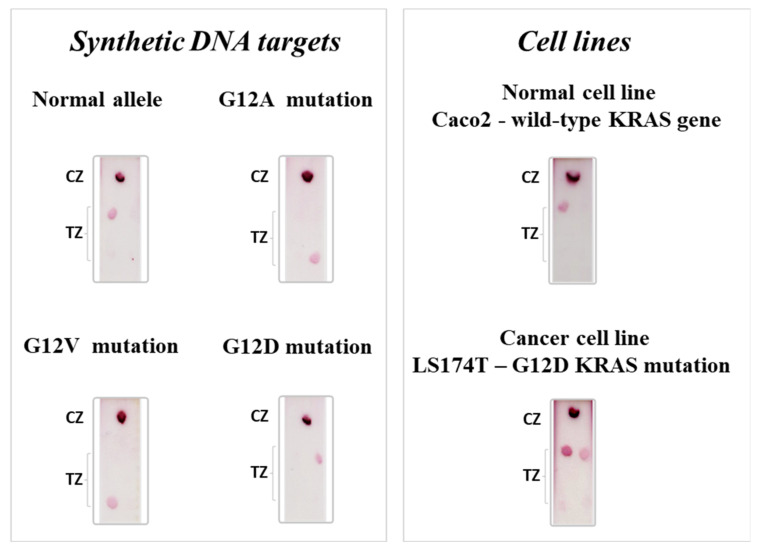
Results of the multiplex strip test for the synthetic DNA targets that correspond to the normal KRAS gene and the three single-point mutations examined. Results of the multiplex strip test for the cell lines that express the wild-type KRAS gene and the G12D KRAS mutant allele. TZ: test zone, CZ: control zone.

The synthetic DNA targets were subjected to a multiplex PEXT reaction in the presence of the four PEXT tagged primers. The reaction was conducted for three cycles, using an annealing temperature of 62 °C and 400 fmol of each the synthetic target. All the PEXT products were finally analyzed separately with a single strip test. Each PEXT product was specifically hybridized, and a visible red spot was formed only at the test spot where the complementary anti-tag sequences were immobilized, corresponding to the target allele present in the sample. The images of the strips were scanned using a regular scanner and are presented in Figure 2. We can observe from the images the good specificity of the multi-analyte strip test.

### 3.2. Cell Lines

DNA isolated from wild-type KRAS and mutant KRAS (G12D) human colon cancer cell lines was also used for optimization experiments. The purified DNA from each cell line was subjected to a single multiplex PEXT reaction containing all four PEXT tagged primers. In comparison to the synthetic targets, a more intense colored signal at the test spots was observed at an annealing temperature of 58 °C and a 15-cycle PEXT reaction, using 400 fmol of the PEXT product. This was attributed to the differences in DNA structures. The synthetic DNA targets are single-stranded oligonucleotides increasing the hybridization efficiency, as well as the non-specific interactions, contrary to the double-stranded genomic DNA. Strip test results from the analysis of the DNA derived from the cell lines are presented in Figure 2. The analysis of the wild-type KRAS cell line (Caco2) resulted in the formation of a red spot only at the TZ of the strip corresponding to the normal allele. As for the cancer cell line LS174T, two red spots were formed both for the normal allele and the G12D KRAS mutation. These results also denoted the high specificity of the multiplex strip test.

### 3.3. Tissue Samples

**Figure 3:**
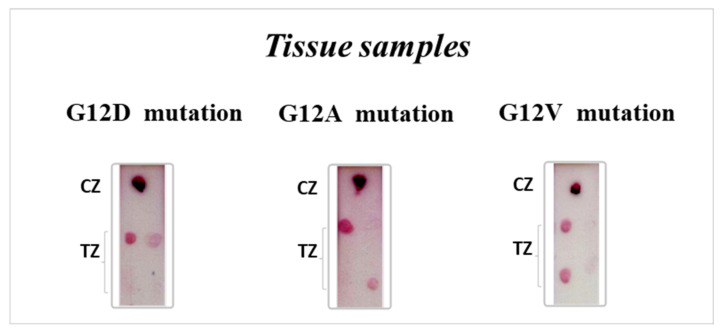
The multiplex strip test results of three tissue samples that have the G12D, G12A, and G12D KRAS mutations. TZ: test zone, CZ: control zone.

The multi-analyte strip-type DNA biosensor was then tested for the detection of KRAS mutations in tissue samples from CRC patients that carried the G12A, G12V, and G12D KRAS gene mutations as previously confirmed by NGS. The optimized parameters required a PEXT reaction of 30 cycles at an annealing temperature of 58 °C, using 200 fmol of the PCR product. The DNA in FPPE tissue samples is partially degraded, leading to a low PCR yield. Thus, only 200 fmol of the PCR product was used for the PEXT reaction, whereas an increase in the cycles of the PEXT reaction was required. The results of the strip test for the tissue samples are shown in Figure 3. Again, only the red spots at the test zones that corresponded to the mutations present in the tissue samples were formed at the membrane of the strips, ensuring the good specificity of the test.

### 3.4. Detectability of the Method

**Figure 4:**
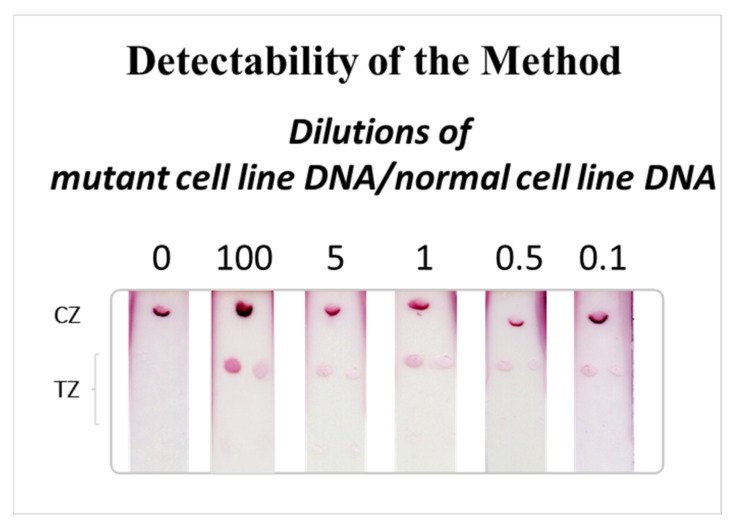
Detectability of the method. Different dilutions of mutant cancer cell line DNA/normal cell line DNA (0.1–100%) were prepared and analyzed by the multiplex strip test after amplification with subsequent multiplex PEXT reaction. TZ: test zone, CZ: control zone.

The detectability of the method, in terms of the input DNA prior to the PCR, was determined as follows. Different dilutions (0, 0.1, 0.5, 1, 5, and 100%) of the DNA isolated from the human colorectal cancer cell line LS174T that carries the mutated (G12D) KRAS allele within the context of unmutated DNA isolated from the human cell line Caco2 (normal KRAS) were prepared. In more details, 0.1, 0.5, 1, and 5 ng DNA from the LS174T cell line were mixed with DNA from normal Caco2 cell line to obtain a total amount of 100 ng for all mixtures. A total of 100 ng of the DNA admixtures, as well as 100 ng of the DNA from the KRAS mutant cell line, were then amplified by PCR. Subsequently, 400 fmol of each PCR product was subjected to multiplex PEXT reaction and 5 μL of the PEXT products were analyzed with the multiplex strip test. The results are presented in Figure 4. As observed, 0.1% (0.1 ng) of the DNA form the KRAS mutant cell line was detectable in the background of normal KRAS DNA. However, as LS174T cell line carries both mutated KRAS and normal KRAS allele, meaning that the amount of mutated KRAS is even smaller, we conclude that less than 0.1% or 0.1 ng of the mutated KRAS gene is detectable by the multiplex strip test.

### 3.5. Application of the Multiplex Rapid Strip Test to Blood Samples for the Detection of KRAS Mutations in cf/ctDNA

**Figure 5:**
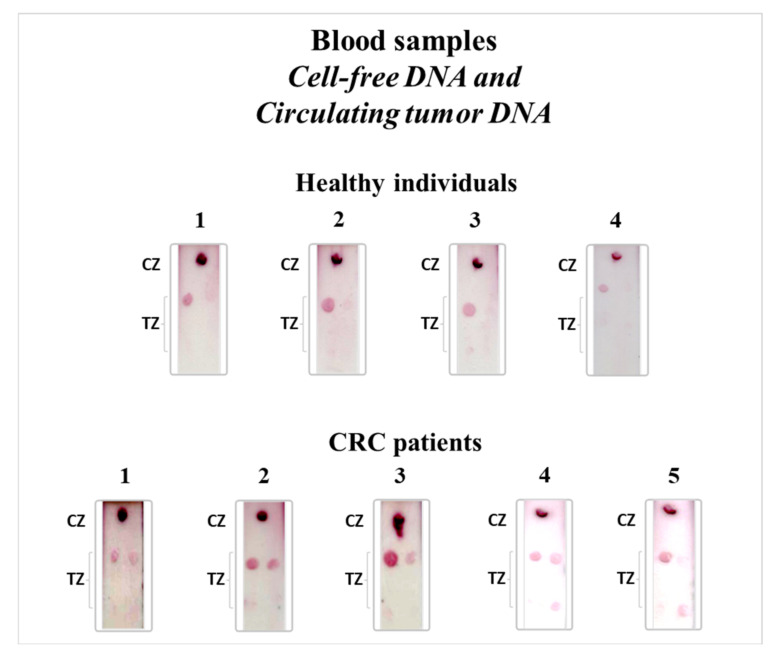
Application of the multiplex strip test for the detection of cell-free DNA and circulating tumor DNA in blood samples of four healthy individuals and five CRC patients. CRC: colorectal cancer, TZ: test zone, CZ: control zone.

The multiplex strip test was finally evaluated for the detection of KRAS gene mutations in blood samples. For this study, four healthy volunteers and five CRC patients with known KRAS mutations previously confirmed by NGS in tissue samples were used and analyzed by the multiplex strip test. The cf DNA was isolated from the blood samples and underwent the multiplex PEXT reaction in the presence of all four allele-specific PEXT primers. As ctDNA is only a small fraction (<1%) of the cfDNA and KRAS gene is therefore present in extremely low abundancies in blood circulation, a 25-cycle PEXT reaction at 58 °C using 200 fmol of the isolated cfDNA was needed in order to successfully detect normal and mutant KRAS gene in blood samples with high specificity. The PEXT products were directly applied to the multiplex strip test according to the established protocol. For healthy individuals, a red spot appeared at the TZ of the biosensor only for the normal allele. On the other hand, for CRC patients with known KRAS mutations, two red spots were formed at the TZ of the strip test that corresponded to the normal allele and to the corresponding KRAS mutation, as expected (Figure 5) with 100% concordance between our assay in plasma and tissue results by NGS. Moreover, we observed, as shown in Figure 5, that the color intensity of the test spot signal for the gene mutations was lower than the signal of the test spot for the normal allele, and this is due to the fact that ctDNA is present at much lower concentration than the normal cfDNA in blood circulation.

### 3.6. Repeatability of the Multiplex Rapid Strip Test

**Figure 6:**
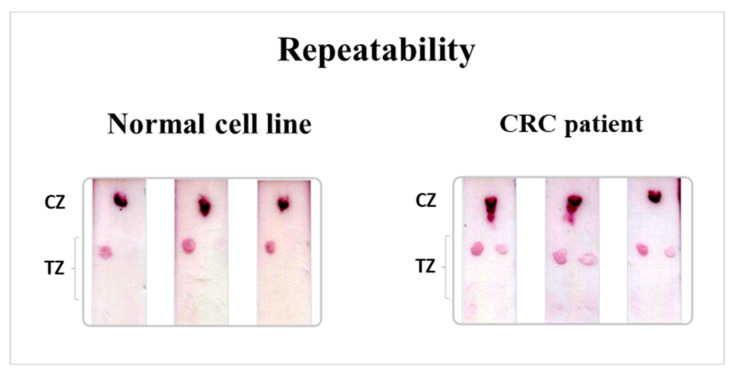
Repeatability of the multiplex strip test for the normal cell line and the blood sample of a CRC patient. TZ: test zone, CZ: control zone.

The repeatability of the developed multiplex strip test was also evaluated and was expressed as % coefficient of variation (%CV) values. For this purpose, isolated DNA (400 fmol) from the wild-type KRAS cell line Caco2 and cfDNA (200 fmol) isolated from blood sample of a CRC patient with a G12D KRAS mutation were subjected to the multiplex PEXT reaction and analyzed in triplicate with the multiplex strip test. The results are presented in Figure 6. The strips were acquired with a conventional scanner and the color density of the red spots at the TZ of the strip was measured in grayscale using the open-source ImageJ image processing software. Finally, the %CVs (*n* = 3) were calculated from the gray values of the test spots of the strip images and were 2.8% for the cell line, 0.5% for the normal allele, and 0.7% for the G12D mutation of the cf/ctDNA from the patient, ensuring the very good repeatability of the multi-analyte strip test.

## 4. Discussion

Biosensors are the most preferable analytical tools compared to conventional analytical methods for ctDNA analysis or bioanalysis in general, because they offer simplicity, low cost, rapid analysis (in some cases), portability, and multiplicity, along with high detectability, sensitivity, specificity, robustness, and reproducibility. They are also easy to use [29]. Moreover, the use of nanomaterials in such devices emerged as an effort to increase the analytical performance of the biosensors, as these nanomaterials provide signal enhancement without the need for enzyme-assisted amplification [30,31]. Currently, there is still the need for the development of novel biosensors for ctDNA detection and liquid biopsy applications.

Herein, we developed a multiplex strip-type biosensor based on gold nanoparticles for visual simultaneous detection by the naked eye of four alleles, wt and G12D (35G>A), G12V (35G>T), G12A (35G>C) mutations of the KRAS gene, that are frequently observed in CRC patients and guide treatment decisions. The test was optimized using single-stranded synthetic DNA targets and cell lines expressing the wild-type and the mutated (G12D) KRAS gene and also allowed for the detection of three different KRAS mutations in FFPE tissue samples from CRC patients. Importantly, our multiplex strip assay could efficiently and with specificity detect different single point mutations of codon 12 of the KRAS gene in cfDNA of five CRC patients with 100% concordance with tissue results previously obtained by NGS, while only the wt allele was detected in healthy individuals. The protocol includes cfDNA isolation, KRAS gene amplification, allele discrimination by a multiplex PEXT reaction, and simultaneous visual detection of the four alleles by a single strip test. Analysis is completed within 10 min, while the run time is approximately 3.5 h. The strip test shows an LOD ≤ 0.1 ng (1:1000 dilution) of mutated KRAS gene as it allowed for the detection of lower than 0.1% or 100 pg mutated KRAS gene in the presence of normal KRAS gene, shows repeatable results, and also has the advantages of portability and universality, as the protocol prior to detection can be easily modified in order to target other gene mutations of interest.

Although many biosensors have been reported in the literature, only a few have been evaluated for ctDNA genotyping in clinical samples, rendering our assay a novel promising tool for liquid biopsy applications. Most of these biosensors are electrochemical due to their high detectability and ease of use. An electrochemical biosensor was developed for the detection of two mutations of the EGFR gene. The detection was based on an amplification refractory mutation system (ARMS) and linear-after-the-exponential PCR to obtain asymmetric biotinylated PCR products. The products were captured to a tetrahedral DNA nanostructure-decorated electrode and detected by avidin-HRP (horseradish peroxidase). This biosensor needs an overnight fabrication and has a >1 h analysis time, while it offers a limit of detection (LOD) of 30 pg, good specificity, and universality potential [32]. An electrochemical biosensor has also been previously developed for the detection of the PIK3CA E545K mutation in ctDNA. The signal of this biosensor was enhanced through hybridization chain reaction (HCR) that took place on a gold electrode’s surface, while the HCR products were biotinylated and detected by streptavidin-alkaline phosphatase through biotin–streptavidin interaction. The sensor’s characteristics were >1.5 h of analysis time, about 17 h of fabrication time, quite good specificity, and LOD of 3 pM, but no multiplexing or universality potential [33]. In an effort to enhance the hybridization efficiency and specificity, Das et al., (2019 and 2016) developed an electrochemical sensor that uses combinatorial DNA probes or clutch probes and PNA clamps. The analyzed ctDNA was specifically captured onto the electrode by immobilized capture DNA probes. The electroactive compounds [Ru(NH_3_)_6_]^3+^ and [Fe(CN)_6_]^3−^ were intercalated to the DNA sequences through electrostatic forces, increasing the sensor’s response. The authors could simultaneously detect seven different mutations of the KRAS gene and several mutations of the EGFR gene with high specificity. The LOD was 1 fg/μL and the analysis time was about 2 h, while an overnight incubation was needed to construct the sensing electrode [34,35]. Another electrochemical biosensor was reported for the dual detection of mutations and epigenetic methylation of ctDNA. The ctDNA was captured by complementary PNA probes that were coupled to gold nanoparticles. Lead phosphate apoferritin conjugated to anti-5-mC was then bound to the methylation sites of ctDNA, forming a sandwich complex on the electrode. Lead ions were finally released from apoferritin and were detected by square-wave voltammetry. Two mutations of the PIK3CA gene were separately detected by this biosensor with high specificity in >1 h, while the biosensor needs about 30 min to be constructed [36]. A dual-signal amplification system was also reported. This system exploited target recycling through RNase HII action, while DNA dendritic nanostructures were synthesized by terminal transferase, increasing the signal. The electrical signal was generated by the electroactive compound methylene blue. This sensor offered high detectability and specificity for the detection of a single KRAS mutation in ctDNA. The sensor could be universal, while the analysis and fabrication time were about 3 and 4.5 h, respectively [37]. Screen-printed multi-carbon electrodes were also used in a single and multiplex format for ctDNA analysis. Capture DNA probes were immobilized onto the electrode’s surface, while the hybridization of the target was performed through ruthenium redox mediator and cyclic voltametric measurements. The fabrication time of the sensor is about 1 h, the analysis time is 3.5 h, while the LOD is 4 copies/ng for the single and 0.58 ng/μL for the multiplex format [37,38]. An urchin-like gold nanocrystal-multiple graphene aerogel was also exploited for ctDNA analysis. In this approach, signal enhancement was accomplished via DNA-induced target recycling. This biosensor had an LOD of 0.033 fM, >1 h analysis time with quite good specificity, and about 4 h fabrication time, but it lacked multiplicity and universality [39,40] RNase HII-aided target recycling was also performed in another SERS assay to detect single-stranded ctDNA in blood samples. T-rich DNA sequences produced by the RNAse were captured on the sensor’s surface. Single-walled carbon nanotubes were then attached to the hybrids, while copper nanoparticles were synthesized to the T-rich areas, increasing the SERS signal. Two mutations of the KRAS and PIK3CA were detected within more than 30 min with high specificity. The biosensor needs extensive fabrication (about 54 h) and does not apply for multiplex or universal analysis [41]. Biosensors based on surface-enhanced Raman spectroscopy (SERS) also emerged in the ctDNA analysis. Multiplex detection of ctDNA mutations was also accomplished by a SERS assay. ctDNA was amplified by an allele-specific PCR. The amplified products carried a biotin moiety at one end and contained overhanging sequences at the other end. The products were captured by streptavidin-coated magnetic beads and detected by complementary DNA probes coupled to multicolor SERS nanotags (AuNPs) through hybridization with the overhanging ends. The hybrids were finally analyzed, after magnetic separation, by a portable Raman microscope. With this system, three mutations of the KRAS, BRAF, and NRAS gene were detected within 30 min. The sensor is universal and offers good specificity and sufficient multiplexing potential [42,43]. In another SERS approach, ctDNA was also amplified by allele-specific PCR. The amplified products were detected by positively charged gold/silver nanostars that were electrostatically bound to the negative DNA sequences. The SERS-active compound 2,3,5,6-tetrafluoro-4-mercaptobenzoic acid was used for SERS signaling. SERS spectrum was obtained by a portable Raman microscope. This assay is a mix-and-read method and applied for the detection of one single-point mutation of the BRAF gene. It offers universality, good specificity, and quite good multiplexing potential [44]. The combination of Fe and Au nanoparticles was also used for the detection of ctDNA. Magnetic DNA1-modified amorphous Fe^0^ nanoparticles were used to magnetically separate and enrich ctDNA sequence, while the detection is accomplished through DNA2-modified Au nanoparticles and subsequent ICP-MS analysis. The method gave an LOD of 0.1pg/mL [45]. A localized surface plasmon resonance (LSPR) was also developed for ctDNA detection of the KRAS gene. ctDNA was captured on the surface of the sensor by PNA-functionalized gold nanorods. The LOD of this sensor is 2 ng/mL [46]. Table 2 and Table S3 contain a comparison between our work and other biosensors and conventional methods that have been applied for ctDNA analysis in clinical samples. Compared to the previously reported biosensors, our proposed multiplex rapid strip test was efficiently applied for cfDNA analysis of CRC patients’ samples and shows great advantages related to specificity and repeatability (%CV < 3%), cost effectiveness, simplicity and ease of use, rapid analysis and fabrication time, portability, universality, and advanced multiplexing potential.

As for lateral flow assays for KRAS gene mutations detection in general, a lateral flow assay based on allele-specific PCR and hybridization to oligonucleotide-decorated AuNPs was developed for the detection of KRAS mutations in genomic DNA isolated from a cancer cell line, but it was not applied for ctDNA analysis [47]. Additionally, a commercially available strip test, the KRAS Strip Assay kit, was used by Kader et al., 2013 for the detection of KRAS mutations in tissue samples from CRC patients. This assay is based on an alkaline phosphatase-catalyzed chromogenic reaction and allele-specific hybridization onto the strip at high temperature (45 °C) to obtain the required specificity. Moreover, the test involves many washing and incubation steps with a total analysis time of about 2 h [48]. Compared to the previously reported biosensors for ctDNA detection in real samples (Table S3) [49,50,51,52,53,54,55,56,57,58,59,60,61,62,63,64,65,66,67,68,69,70,71,72,73,74,75,76,77,78,79,80,81], the advantages of the proposed multi-plex test are related mostly to the cost, run time, and simplicity, as well as the universality and advanced multiplexing potential.

Albeit the great advantages of the developed test, it should be noted that there are some limitations. Although no false-positive or false-negative results were obtained when the test was applied in cfDNA of healthy donors and CRC patients with 100% concordance with the tissue results previously confirmed by NGS, the number of patient samples used in the study for validation is relatively low. Therefore, further studies with a larger series of CRC patients and comparison with another technique, such as droplet digital PCR or NGS, are required to evaluate the clinical sensitivity and specificity of this newly developed biosensor as a diagnostic tool in liquid biopsy applications. In this context, a receiver operating characteristic (ROC) curve analysis of the results obtained by the two methods would be an effective approach to evaluate the sensitivity and specificity of our assay for diagnostic purposes [82]. Moreover, the multiplex strip test presented herein shows LOD ≤ 0.1 ng (1:1000 dilution) of mutated KRAS gene which is comparable with other methods such as ddPCR [82} and most importantly efficiently and specifically detected KRAS wt or mutant alleles in plasma samples with cfDNA concentrations as low as 6 ng/μL. However, considering that ctDNA is a challenging analyte due to the low concentration and high fragmentation and the amount of input DNA is a critical step in developing methods suitable for cfDNA analysis, further optimization in this context would be valuable.

## 5. Conclusions

In conclusion, we have developed a rapid and low-cost multiplex strip test for genotyping of cell-free/circulating tumor DNA in blood samples. The detection was based on gold nanoparticles. This newly developed strip test was applied for multiplex detection of KRAS mutations in liquid biopsies of CRC patients. Liquid biopsy represents a competing non-invasive tool for tumor genotyping, providing valuable information employed for cancer diagnosis, prognosis, and guidance of treatment decisions. Several methodologies have been used to detect tumor-specific aberrations in ctDNA, many of which are complicated, expensive, and time-consuming. The proposed method is a non-invasive, simple, and low-cost approach for the rapid and accurate analysis of cell-free and circulating tumor DNA. It achieves simultaneous multi-allele detection with a single test with high specificity and repeatability (%CV < 3%), offers an LOD lower than 0.1% (100 pg) mutated KRAS gene, and avoids multiple washing and incubation steps. It is also universal, meaning that it can be applied for the detection of any gene mutation or single nucleotide polymorphism (SNP), since the discrimination of the alleles takes places before the analysis with the strip test. The test was initially developed using DNA from synthetic DNA targets, cancer cell lines, and tissue samples. The proposed strip test was finally applied, for the first time, for the multiplex detection of KRAS mutations in cfDNA and ctDNA isolated from plasma samples from healthy individuals and CRC patients. All four KRAS alleles in ctDNA were simultaneously detected with a single strip test. The visual detection with the multiplex strip test was completed within 15 min, while the whole protocol, including DNA purification, KRAS gene amplification, allele-discrimination reaction, and the multiplex strip test, was completed within 3.5 h. Although further studies are required to validate the clinical sensitivity and specificity of the test, the results obtained so far render this test a promising tool for future liquid biopsy applications.

## Figures and Tables

**Table 1:** Sequences of the PCR and PEXT primers, the synthetic DNA targets, and the anti-tag sequences attached onto the polystyrene microspheres.

Oligonucleotide	Sequence (5′ → 3′)
**PCR Primers**	
KRAS_Forward	GCCTGCTGAAAATGACTGAATA
KRAS_Reverse	CAAGAGACAGGTTTCTCCATCA
**Synthetic Targets**	
KRAS-Normal	CTGAATTAGCTGTATCGTCAAGGCACTCTTGCCTACGCCACCAGCTCCAACTACCACAAG
KRAS-G12D	CTGAATTAGCTGTATCGTCAAGGCACTCTTGCCTACGCCATCAGCTCCAACTACCACAAG
KRAS-G12V	CTGAATTAGCTGTATCGTCAAGGCACTCTTGCCTACGCCAACAGCTCCAACTACCACAAG
KRAS-G12A	CTGAATTAGCTGTATCGTCAAGGCACTCTTGCCTACGCCAGCAGCTCCAACTACCACAAG
**Anti-Tag Sequences**	
KRAS-NORMAL	NH_2_-GGATACCGCTGCACCCATCGCCAC
KRAS-G12D	NH_2_-CGTTTTAAGTTCGGATGGTGACGT
KRAS-G12V	NH_2_-AGCGCACTGGTGGATGCTGGACTG
KRAS-G12A	NH_2_-CTTGCTGAACTTCTGACTACGACT
**Tag-PEXT Primers**	
KRAS-NORMAL	GTGGCGATGGGTGCAGCGGTATCCCCGAATTCTCTCCTTGTGGTAGTTGGAGCTGG
KRAS-G12D	ACGTCACCATCCGAACTTAAAACGCCGAATTCTCTCCTTGTGGTAGTTGGAGCTGA
KRAS-G12V	CAGTCCAGCATCCACCAGTCGGCTCCGAATTCTCTCCTTGTGGTAGTTGGAGCTGT
KRAS-G12A	AGTCGTAGTCAGAAGTTCAGCAAGCCGAATTCTCTCCTTGTGGTAGTTGGAGCTGC

**Table 2:** Comparison of biosensors for gene mutations detection in ctDNA in real samples.

Gene	Method	Fabrication Time	Analysis Time (after Amplification)	LOD	Precision (%CV)	Multiplicity	Universality	Ref.
EGFR	HPR- and DNA nanostructure-based electrochemical biosensor	overnight	>1 h	30 pg	1.89	2	✓	[31]
PIK3CA	Alkaline-phosphatase and HCR-based electrochemical biosensor	17 h	>1.5 h	3 pM	-	-	-	[32]
KRAS EGFR	Electrochemical sensor	Overnight	>30 min	1 fg/μL	-	array of 40 sensors	-	[33,34]
PIK3CA	Electrochemical platform	>1 h	1 h	10 fM	5.3	-	-	[35]
KRAS	Triple-helix molecular switch-based electrochemical biosensor	>4.5 h	3 h	2.4 aM	5.5–7.4	-	✓	[36]
KRAS	DNA probe-functionalized electrochemical sensor	1 h	3.5 h	4 copies/ng	-	-	-	[37]
KRAS	DNA probe-functionalized electrochemical sensor	1 h	3.5 h	0.58 ng/μL	-	3	-	[38]
KRAS	Urchin-like gold nanocrystal-multiple graphene aerogel	>4 h	>1 h	0.033 fM	-	-	-	[39]
KRAS	RNase assisted SERS platform	58 h	-	0.3 fM	-	-	✓	[40]
BRAF, NRAS	PCR/SERS sensor	>48 h	-	10 copies	8.8	3	✓	[41]
KRAS, BRAF	PCR/ SERS sensor	-	~30 min	-	-	3	-	[42]
BRAF	PCR/SERS sensor	>50 min	-	100 input copies	4	2	-	[43]
KRAS	Fe–Au nanoparticle-coupling/ICP-MS	-	-	0.1 pg/mL	-	7	-	[44]
KRAS	PNA probes on gold nanorods	-	10 min	2 ng/mL	-	-	-	[45]
KRAS	Strip-type biosensor	10 min	10 min	50 amol(10 pM) of ssDNA or <0.1% (100 pg) mutated gene	0.5–2.8	4	✓	This work

HPR: horseradish peroxidise. CP: combinatorial probes. SERS: surface-enhanced Raman spectroscopy. HCR: hybridization chain reaction. ICP-MS: inductively coupled plasma mass spectrometry. PNA: peptide nucleic acid. TMSDR: toehold-mediated strand displacement reaction.

## Supplementary Materials

The following supporting information can be downloaded at: https://www.mdpi.com/article/10.3390/bios12020097/s1, Table S1: Information for the CRC patients. Supplementary Table S2. The quantification results for DNA isolation from the cell lines and ctDNA isolation from the blood samples. Table S3: Conventional methods for the detection of KRAS mutations in ctDNA isolated from blood samples. Figure S1. Electropherograms of the PCR products obtained from DNA and ctDNA from a cell line, a tissue sample and plasma blood samples from CRC patients. M: DNA marker, CRC: colorectal cancer. Figure S2. Evaluation of SA-AuNPs conjugates. b-BSA: biotinylated bovine serum albumin. Figure S3. Optimization study for the sample volume for the analysis with multiplex rapid strip test. Figure S4. Calibration graph for the detection of single-stranded (ssDNA) with the rapid strip test. CZ: control zone, TZ: test zone, b-dA30: biotinylated dA30 oligonucleotide.
