# Supplementary material for: Rapid Multiplex Strip Test for the Detection of Circulating Tumor DNA Mutations for Liquid Biopsy Applications
Source: Biosensors (Basel). 2022 Feb 4;12(2):97. doi: 10.3390/bios12020097 (PMC8869478; doi:10.3390/bios12020097)
Supplement: Supplementary file 1 [file biosensors-12-00097-s001.zip › biosensors-1530533-supplementary.pdf]

biosensors MDPI

Supplementary Material

Rapid multiplex strip test for the detection of circulating tumor DNA mutations for liquid biopsy applications

Kalligosfyri Panagiota[1#], Nikou Sofia[2#], Karteri Sofia[3], Kalofonos P. Haralabos[3], Bravou Vasiliki[2, *] and Kalogianni P. Despina[1, *]

[1]*Department of Chemistry, University of Patras, 26504 Rio, Patras, Greece*

[2]*Department of Anatomy-Histology-Embryology, Medical School, University of Patras, 26504, Patras, Greece*

[3]*Division of Oncology, Department of Internal Medicine, University Hospital of Patras, 26504, Rio, Greece.*

# equal contribution

***Corresponding author:**

Despina P. Kalogianni, Assistant Professor

E-mail address: kalogian@upatras.gr

Vasiliki Bravou, Pathologist, Associate Professor

E-mail address: vibra@upatras.gr

Supplementary Table S1. Information for the CRC patients.

Patient	Gender	Age	KRAS mutation	Stage	Chemotherapy Treatment at time of sample collection	Sample type
1	Female	71	G12D	III	Yes	Blood
2	Male	65	G12D	IV	Yes	Blood & FFPE tissue
3	Male	73	G12D	IV	No	Blood
4	Male	64	G12A	IV	No	FFPE tissue
5	Female	69	G12V	IV	No	FFPE tissue
6	Female	74	G12D,A	IV	Yes	Blood
7	Female	70	G12A	IV	Yes	Blood

FFPE: formalin-fixed paraffin-embedded.

Supplementary Table S2. The quantification results for DNA isolation from the cell lines and ctDNA isolation from the blood samples.

Samples	Concentration (ng/uL)
Cell lines	
wild-type KRAS cell line Caco2	100
mutant KRAS cell line LS174T	10
FFPE tissue samples	
G12D mutation	37.45
G12A mutation	30.65
G1V mutation	370
Healthy individuals	
H1	6
H2	6
H3	10
CRC patients	
P1 (G12D mutation)	110
P2 (G12D mutation)	10
P3 (G12D mutation)	10
P4 (G12D and G12A mutation)	7.5
P5 (G12A mutation)	8

1. Methods

1.1. Biotinylation of bovine serum albumin

The biotinylated bovine serum albumin (b-BSA) with a final concentration of 0.8 mg mL^{-1} was prepared as previously reported [27].

2. Results

2.1. KRAS gene amplification

KRAS gene was amplified by PCR from DNA isolated from the cell lines and ctDNA isolated from plasma blood samples. The PCR products were analyzed by agarose gel electrophoresis and ethidium bromide staining. The size of the PCR products was 171 bp. A typical electropherogram is shown in Figure S1.

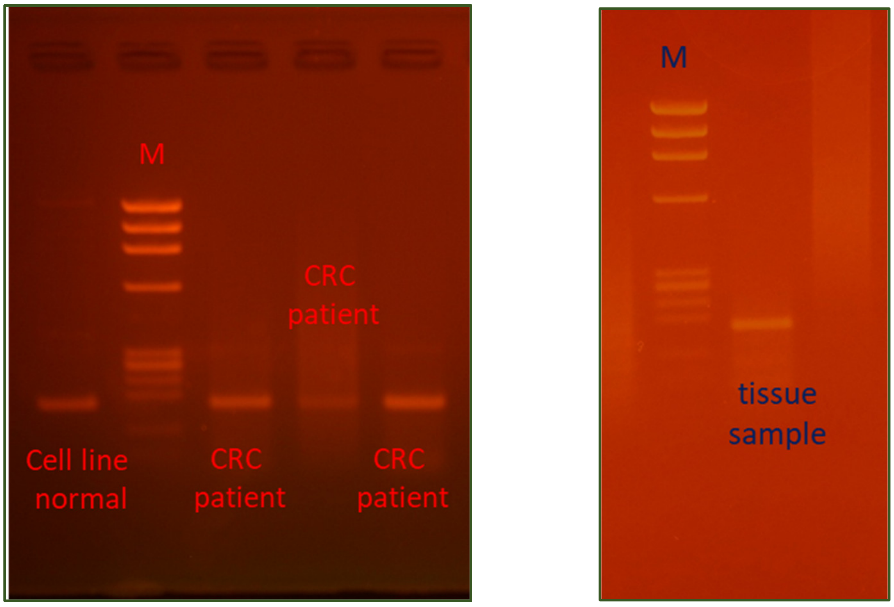

Figure S1. Electropherograms of the PCR products obtained from DNA and ctDNA from a cell line, a tissue sample and plasma blood samples from CRC patients. M: DNA marker, CRC: colorectal cancer.

2.2. Evaluation of SA-AuNPs conjugates

The preparation of the SA-AuNPs conjugates was evaluated by testing the specific capturing to biotinylated BSA onto the membrane of the strip test compared to non-biotinylated BSA. The results are presented in the following Figure. We observed that the SA-AuNPs were captured only at the area of the membrane where the b-BSA was immobilized due to streptavidin-biotin interaction.

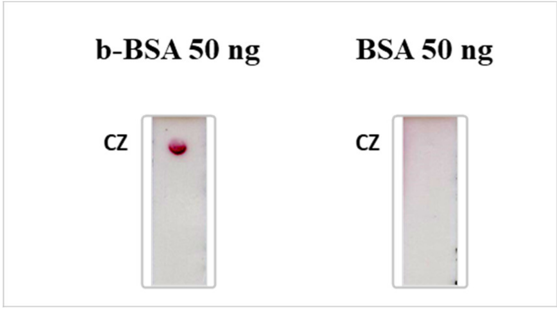

Figure S2. Evaluation of SA-AuNPs conjugates. b-BSA: biotinylated bovine serum albumin.

2.3. Optimization of the sample volume for the multiplex rapid strip test

The sample applied onto the conjugation pad of the strip test was also optimized. For this purpose, different volumes (1-10 μL) of the PEXT product obtained from the cell line that expresses the wild-type KRAS gene were analyzed with the multiplex strip test. We observed that the optimum sample volume of the product was that of 5 μL (Figure S2).

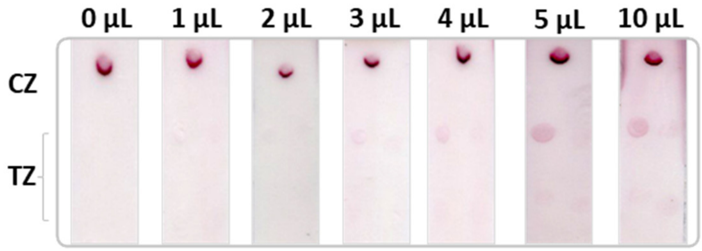

Figure S3. Optimization study for the sample volume for the analysis with multiplex rapid strip test.

2.4. Evaluation of the analytical performance of the rapid strip test

In order to determine the detectability of the proposed strip test, we tested the analytical performance of a single-stranded DNA (ssDNA) that carries a biotin moiety at one end (b-dA_{30}). Carboxylated polystyrene microspheres conjugated to an NH_2-dT_{30} DNA probe were immobilized onto the membrane to construct the test zone of the strip. Biotinylated BSA was also used to form the control spot. Finally, a calibration graph using different amounts (0-50 fmol) of the ssDNA was constructed using the strip test. The results are presented in Figure S3. As low as 50 amol (in 5 μL sample volume) of ssDNA were detectable by bare eye with the strip test, assuring the very good detectability of the test.

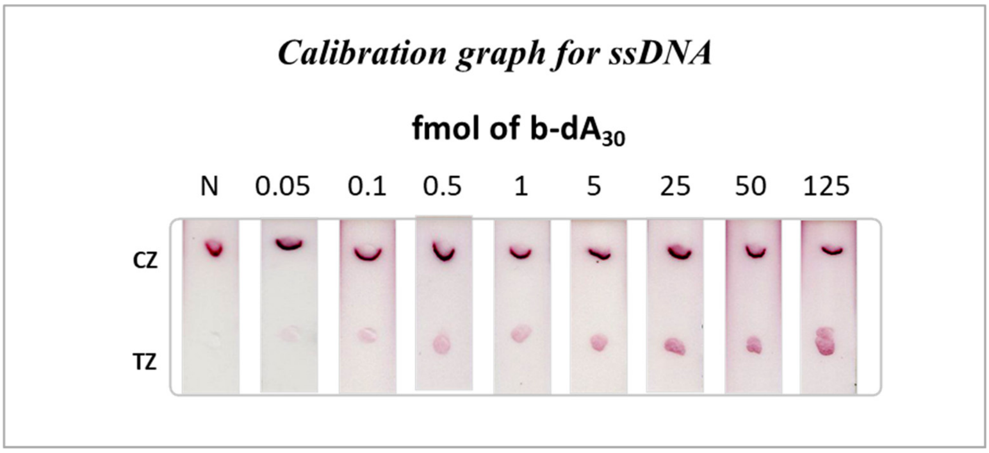

Figure S4. Calibration graph for the detection of single-stranded (ssDNA) with the rapid strip test. CZ: control zone, TZ: test zone, b-dA_{30}: biotinylated dA3$_0$ oligonucleotide.

Supplementary Table S3: Conventional methods for the detection of KRAS mutations in ctDNA isolated from blood samples.

Method	Total analysis time	LOD	Multiplicity	Universality	Ref
real-time PCR	<3 h	0.5-5%	-	✓	[49]
Idylla Platform (real-time PCR)	2h 10 min	0.01-0.1%	-	✓	[50]
allele-specific real-time PCR	-	1%	28	-	[51]
ARMS- PCR	80 min	0.2%	-	✓	[52]
mePCR-qDMA	<40 min	0.1% and 6 %	2	-	[53]
PCR-LDR-qPCR assay	-	2-5 mol	5	-	[54]
digital PCR	>3 h	-	31	✓	[55]
digital PCR	>3 h	0.166% -2.638%	-	✓	[56]
digital PCR	<3 h	0.3%	10	-	[57]
ddPCR	>2h	-	-	-	[58]
ddPCR	>2h	0.09%	4	-	[59]
ddPCR	>2h	12-22 copies	3	-	[60]
IC3D dPCR	>2 h	0.005%	2	✓	[61]
ddPCR	>2 h	0.2%	12	-	[62]
ddPCR	>2 h	1%	13	✓	[63]
ddPCR	<2h	0.2%	7	-	[64]
ddPCR	-	-	14	-	[65]
ddPCR	70 min	<0.1%	8	-	[66]
ddPCR	80 min	0.006 ng/uL	6	-	[67]
ddPCR	>4h	-	14	-	[68]
ultra-deep NGS	-	0.14%	37	✓	[69]
NGS	-	5%	9	✓	[70]
hybrid capture-NGS	-	-	-	-	[71]
dNGS with MBs	-	0.2%	50	✓	[72]
NGS	-	0.1%	-	-	[73]
ultra-deep MPS	80 min	<1%	-	✓	[74]
ultra-deep MPS	-	1%	6	-	[75]
in-tube hybridization and universal tag-microarray	<90 min	0.03 -0.28%	22	✓	[76]
toehold-mediated strand displacement reaction/FRET	3h	6.3 pM	-	✓	[77]
SCC-MAG method	>3 h	2.25 pg/ml	-	-	[78]
switch-blocker technology	>2h	0.01–0.02%	2	-	[79]
nCounter SNV panel hybridization-based platform	18-24 h	0.02–2%	97	-	[80]
NAVIGATER assay	1h	<0.01%	3	-	[81]
Strip-type biosensor	3.5 h	50 amol (10 pM) or <0.1% (100 pg) mutated KRAS gebe	4	✓	This work

Idylla: Fully-automated, real-time PCR

ARMS: Amplification refractory mutation system

LDR: Ligase Detection Reaction

ddPCR: Droplet digital PCR

IC3D: Integrated comprehensive droplet digital detection

NGS: Next-generation sequencing

dNGS: Digital next-generation sequencing

MBs : Molecular Barcodes

MPS: Massively parallel sequencing

mePCR-qDM: Mutant-enriched PCR - quantitative DNA melting curve analysis

FRET: Fluorescence resonance energy transfer

SCC-MAG: Selective capture of ctDNA on magnetic beads

SNV: Single Nucleotide Variant

NAVIGATER: Nucleic Acid enrichment Via DNA Guided Argonaute from Thermus thermophilus

Percentage LOD: mutant copies per copies of DNA input (0.2 % is 2 mut in 1000 normal) or mutant allele frequency
